# Ultrafast Doppler imaging and ultrasound localization microscopy reveal the complexity of vascular rearrangement in chronic spinal lesion

**DOI:** 10.1038/s41598-022-10250-8

**Published:** 2022-04-21

**Authors:** Benoit Beliard, Chaimae Ahmanna, Elodie Tiran, Kadia Kanté, Thomas Deffieux, Mickael Tanter, Fatiha Nothias, Sylvia Soares, Sophie Pezet

**Affiliations:** 1 Institute of Physics for Medicine Paris, Inserm U1273, ESPCI PSL Paris, CNRS UMR8361, PSL Research University - Paris, 17 rue Moreau, 75012 Paris, France; 2 Neuroscience Paris Seine NPS, CNRS UMR8246, INSERM U1130, UM119, Institut de Biologie Paris Seine IBPS, Sorbonne Université Sciences, Campus UPMC, 75005 Paris, France

**Keywords:** Neuroscience, Imaging techniques

## Abstract

Acute spinal cord injury (SCI) leads to severe damage to the microvascular network. The process of spontaneous repair is accompanied by formation of new blood vessels; their functionality, however, presumably very important for functional recovery, has never been clearly established, as most studies so far used fixed tissues. Here, combining ultrafast Doppler imaging and ultrasound localization microscopy (ULM) on the same animals, we proceeded at a detailed analysis of structural and functional vascular alterations associated with the establishment of chronic SCI, both at macroscopic and microscopic scales. Using a standardized animal model of SCI, our results demonstrate striking hemodynamic alterations in several subparts of the spinal cord: a reduced blood velocity in the lesion site, and an asymmetrical hypoperfusion caudal but not rostral to the lesion. In addition, the worsening of many evaluated parameters at later time points suggests that the neoformed vascular network is not yet fully operational, and reveals ULM as an efficient in vivo readout for spinal cord vascular alterations. Finally, we show statistical correlations between the diverse biomarkers of vascular dysfunction and SCI severity. The imaging modality developed here will allow evaluating recovery of vascular function over time in pre-clinical models of SCI. Also, used on SCI patients in combination with other quantitative markers of neural tissue damage, it may help classifying lesion severity and predict possible treatment outcomes in patients.

## Introduction

Traumatic injuries of the spinal cord (SCI) can lead to life-long loss of sensation and voluntary motor functions. Although injured adult neurons of the mammalian central nervous system (CNS) can initially survive, regrowth of their axons through the lesion ultimately fails due to a cascade of cellular and molecular events leaving the affected neural tissue in a permanently altered, regeneration-inhibited state (for review, see^1–3^). Despite clinical advances in rehabilitation and novel treatments based on neuromodulation that improve their quality of life, patients still suffer from the devastating consequences of SCI, aggravated by physiological and psychological complications. Nevertheless, elucidation of the cellular and molecular mechanisms underlying the complexity of SCI, achieved over the last decades, has raised hope for a future development of clinically applicable therapeutic solutions.

A major complication of SCI pathophysiology is due to injury-associated vascular damage followed by hypoxia, hemorrhage and edema, which accelerate necrosis of the affected neural tissue. The associated breakdown of the blood-spinal cord barrier favors infiltration of blood-derived monocytes/macrophages that, together with activated microglia, spread inflammation beyond the initial lesion site, contributing to secondary expansion of the lesion and increase in neurological deficits (for review, see^4,5^). Therefore, it seems important to develop appropriate biomarkers to better assess the vasculature damage, and the limits of its spontaneous restoration, which still remain not well understood.

The initial mechanical injury of the vascularized spinal cord tissue (for review, see^6^) provokes, in the acute phase, a dramatic death of endothelial cells, and a decrease of the vessel density. One week post-injury, local neo-angiogenesis starts^7–11^, but only part of the newly formed blood vessels become stable and functionally integrated. Therefore, the density of mature vessels remains low in comparison to intact tissue.

Thanks to recent progress in the field of neuroimaging, we now dispose of methods to rapidly and reliably analyze the initial extent of vascular damage on the exposed spinal cord. Thus, using ultrafast Doppler imaging^12–15^ or enhanced ultrasound imaging (involving injection of a contrast agent to enhance sensitivity^16,17^), pre-clinical studies have imaged spinal hemodynamics in intact animals^15^, and its hypoperfusion at early stages after SCI^12,16,17^.

**Figure 1:**
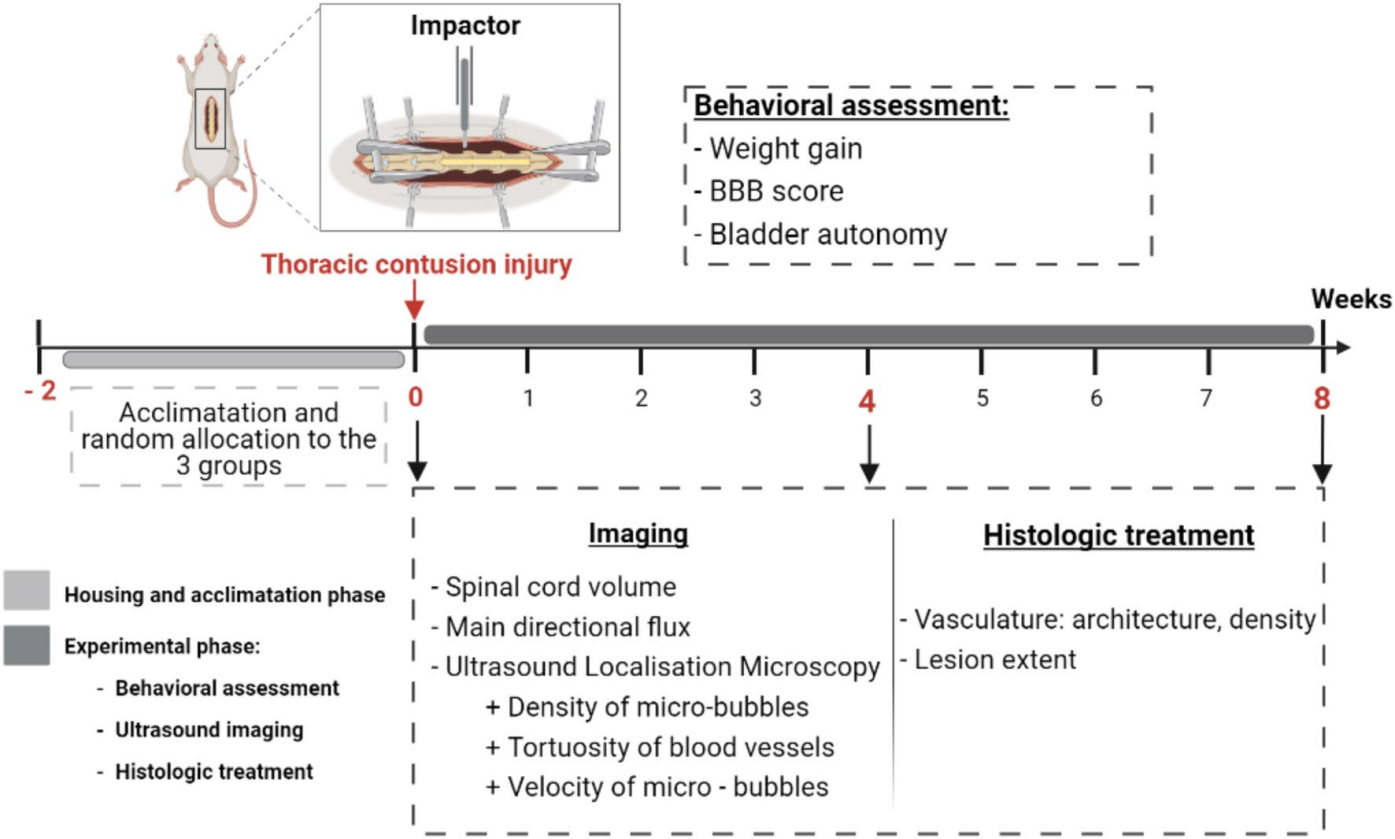
Schematic outline of the experimental design. N = 10 animals received a contusion of the thoracic spinal cord (thoracic levels T8–T9). A behavioral assessment was performed before and once a week after the lesion. At 4 and 8 weeks after contusion, spinal cords of N = 5 animals per group were imaged through an opening created by laminectomy. At the end of the imaging session, animals were cardially perfused in order to perform immunohistological analyses. The top panel was created using Biorender.com.

Combining ultrafast Doppler imaging and ultrasound localization microscopy (ULM) on the same animals, the present study aimed at quantifying trauma-induced alterations of spinal blood volume, main spinal blood flow, density of blood vessels, vessel tortuosity and finally, flow velocity in the various subparts of the vascular network. The study was undertaken at two time points post-injury (Fig. 1), coinciding with restoration of the blood-spinal cord barrier (4 weeks), and with the establishment of the chronic lesion (8 weeks post-lesion)^11,18^. Our study reveals an asymmetric hypoperfusion of the spinal cord associated with reduction of blood flow, alterations of the blood vessel density and morphology, with a worsening at 8-weeks post-contusion.

## Results

### Reduced spinal blood volume within the lesion and alterations of the direction of blood flow in lesioned spinal cord

**Figure 2:**
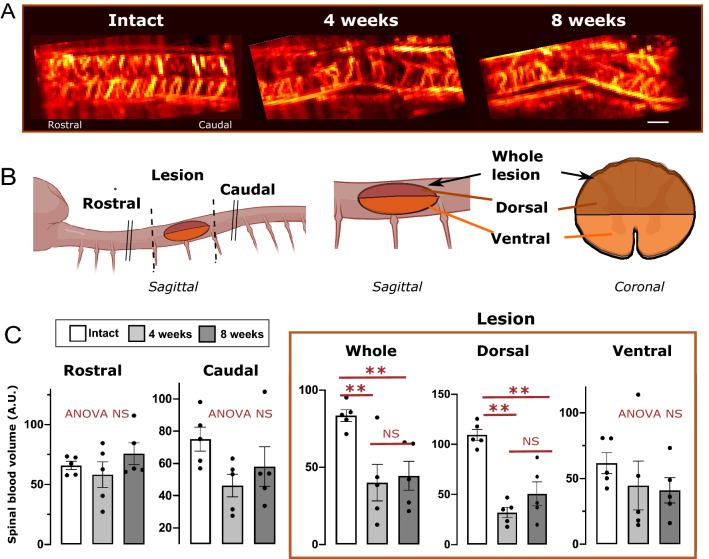
Spinal cord contusion leads to a reduction in spinal blood volume (SBV) in different parts of the lesioned spinal cord at 4- and 8-weeks post-contusion. (**A**) Examples of fUS Doppler images in representative animals. Imaging was performed in the sagittal plane at the level of the midline. (**B**) Cartoons representing the location of SBV analysis rostral or caudal to the lesion, or within the dorsal or ventral part of the lesion site. (**C**) Quantifications show no significant changes in SBV rostral or caudal to the lesion. A clear decrease of SBV is found at the lesion site, attributable exclusively to the dorsal horn. Results are expressed as mean spinal blood volume (Arbitrary units (A.U.) ± SEM, overlaid with individual values. N = 5 animals per groups. Stats: ANOVA, followed by unpaired t-test as post-hoc test. *p < 0.05, **p < 0.01. NS: Not statistically significant, ANOVA NS: ANOVA *p* > 0.05. Bar: 1 mm. Illustrations in the panel (**B**) were created using Biorender.com.

Imaging the cord’s blood volume using ultrafast Doppler imaging (UDI) in a sagittal plane at the level of the midline, with the lesion site being at the center of the imaging plane, allowed for measurements of spinal blood volume (SBV) in different spinal compartments of equivalent surface, at the lesion level, and rostral or caudal to it, in the three groups of animals included in this study. Both at 4- and 8-weeks post-injury, the SBV was strongly reduced at the lesion site, and almost exclusively in the dorsal horn, being non-significant in the ventral horn (Fig. 2C); possibly due to dorso-ventral orientation of the traumatic impact. In addition, although not significant, a tendency of SBV reduction is also noted in caudal segments at both time points post-lesion analyzed, while it was unmodified in rostral segments (Fig. 2A–C).

**Figure 3:**
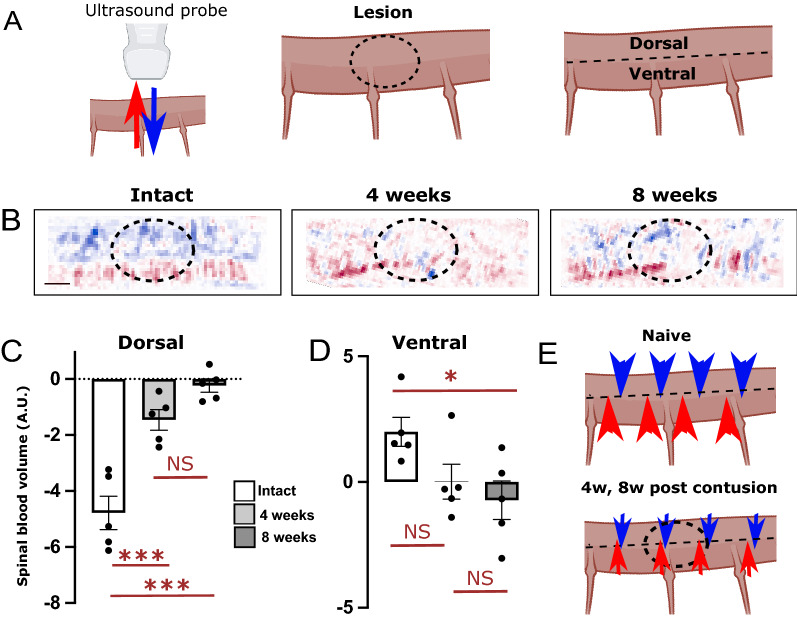
Alterations of mean directional spinal blood volumes over the whole cord 4- and 8-weeks post-contusion. Using a fast sequence of imaging, the main directional flow in the whole dorsal or ventral part of the thoracic cord was quantified. (**A**) Cartoons illustrating the mean directional blood flow (left), the localization of the lesion in the images displayed in (**B**) (middle), and the separation between the dorsal and ventral parts analyzed here (right panel). By convention, a negative flow is directed away from the probe, i.e. dorso-ventrally oriented (**A**, left panel, blue arrow). (**B**) Representative examples of these directional power images in animals of the three groups studied. In intact animals, the main directional blood volume is upward in the ventral horn and downwards in the dorsal horn. At both 4 and 8 weeks post-contusion, these flows are deeply altered. Quantification of these changes (**C**, **D**) show a statistically significant decreased directional flow over the whole dorsal horn (**C**) and a more modest reduction in the ventral horn (**D**). (**E**) Schematic of the results obtained, highlighting the fact that the changes are widespread in the hemi-cords. Results in (**C**, **D**) are expressed as mean spinal blood volume (A.U.) ± SEM and are presented in overlay with individual values. N = 5 animals per groups. Stats: ANOVA, followed by unpaired t-test as post-hoc test. *p < 0.05, ***p < 0.001. NS: Not statistically significant. Bar: 1 mm. Illustrations in the panels (**A**) and (**E**) were created using Biorender.com.

Using a UDI sequence with a higher sample frequency (Pulse Repetition Frequency 20,000 Hz, 5 compounded plane waves resulting in a 4000 Hz frame rate), the blood volumes going toward or away from the probe were separated through spectral analysis, allowing quantification of the main directional blood flow. By convention, a positive value means blood flow in ventro- dorsal orientation (Fig. 3A, red arrow), while a flow in the opposite direction has a negative value (blue arrow).

In intact animals, throughout the whole cord imaged, the main directional blood flow in the dorsal horn is dorso-ventral (Fig. 3A,B blue arrow, flow going away from the probe), while in the ventral horn it is a ventro-dorsal flow (i.e. going towards the probe, red arrow, Fig. 3A,B). Four- and eight-weeks post-contusion, the main directional flow along the spinal cord was altered significantly. Both in the dorsal and ventral horns, the main directional flow was reduced (Fig. 3C,D,E) and disorganized (Fig. 3B). Strikingly, in contrast to the measurements of SBV above, the changes of main flow affected the entire cord imaged, not only the lesion site, with a tendency for a worsening in the ventral horn at 8 weeks, compared to 4 weeks post lesion. Due to the high imaging rate, ultrafast Doppler is able to detect and quantify both high (10–18 mm/s) and slow (1–5 mm/s) blood flows^19^. While both are detected in this imaging plane, it is likely that most of the changes in directional blood flow is due to arterial damage.

### Ultrasound localization microscopy for the study of structural vascular abnormalities and alterations in the speed of blood flow

**Figure 4:**
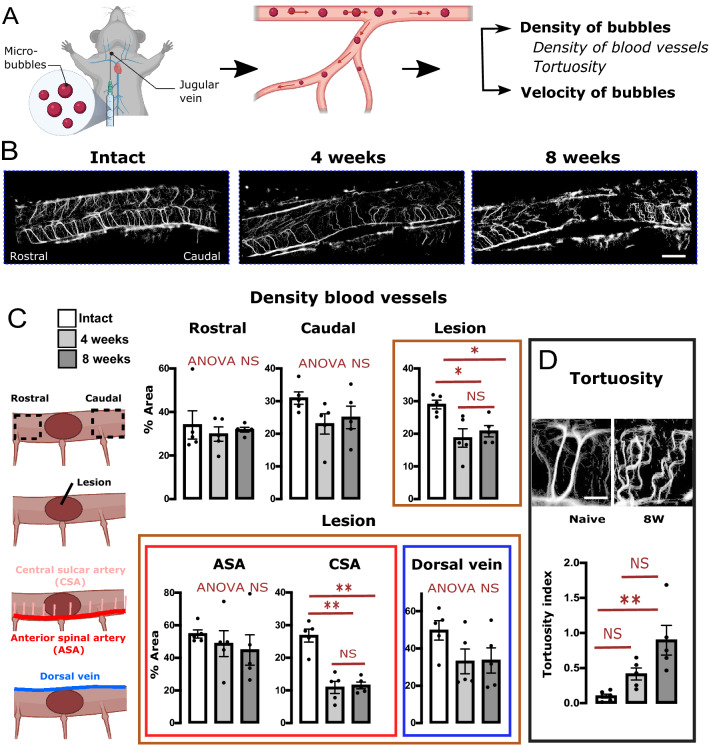
Blood vessel density and tortuosity changes after spinal cord contusion revealed by ULM. (**A**) Schematic of ULM. At the end of the above fUS imaging, 150 μl of contrast agent (bio-compatible microbubbles) were injected intravenously. Using a fast sequence of imaging and further single bubble localization and tracking, this approach allows to determine the normalized density of bubbles and the velocity of the bubbles (which is a proxy for the local velocity of blood flow). (**B**) Representative examples of images of density of micro-bubbles in intact animals, and 4- and 8-weeks post-contusion. (**C**) Quantification of the density of blood vessels in various compartments of the lesioned spinal cord (performed using images of the normalized density of bubbles) reveals loss of blood vessels within the lesion and in central sulcar arteries (CSA). (**D**) Quantification of the tortuosity of the central sulcar artery, using ULM. Top panel: high power magnification images of a intact animal (left) and a lesioned animal (8 weeks post lesion, right), illustrating the tortuosity in CSA. Quantification of this tortuosity shows a time-dependent increased tortuosity in CSA. The orientations (caudal/rostral) apply to the three examples in (**B**). Results are expressed as mean ± SEM and are presented in overlay with individual vales. N = 5 animals per group. Stats: ANOVA, followed by unpaired t-test as post-hoc test. *p < 0.05, **p < 0.01. NS: Not statistically significant. ANOVA NS: ANOVA *p* > 0.05. The orientations (caudal/rostral) apply to the three examples in (**A**). ANOVA NS: ANOVA *p* > 0.05. Bar: (**B**): 1.3 mm, (**D**): 0.4 mm. Illustrations in the panels (**A**) and (**C**) were created using Biorender.com.

ULM is capable of localizing and tracking intravenously injected microbubbles, the trajectory of which will then define the vascular arborization. We then used the individual images generated by this analysis (representative examples from the three experimental groups are shown in Fig. 4B) to analyze the anatomical alterations in the blood vessels, such as changes in density (Fig. 4C) and their tortuosity (Fig. 4D), at the lesion site and at rostrally and caudally adjacent segments. Quantification of the density of blood vessels revealed a strong reduction within the lesion that did not change significantly between 4- and 8-weeks post-lesion (Fig. 4C, brown frame). This effect is attributable to a reduced blood vessel density in the central sulcar arteries (CSA), the only vascular compartment where the reduction was significant (Fig. 4C bottom graphs). Analysis of the tortuosity in these arteries revealed an increase (in tortuosity) with time (Fig. 4D).

**Figure 5:**
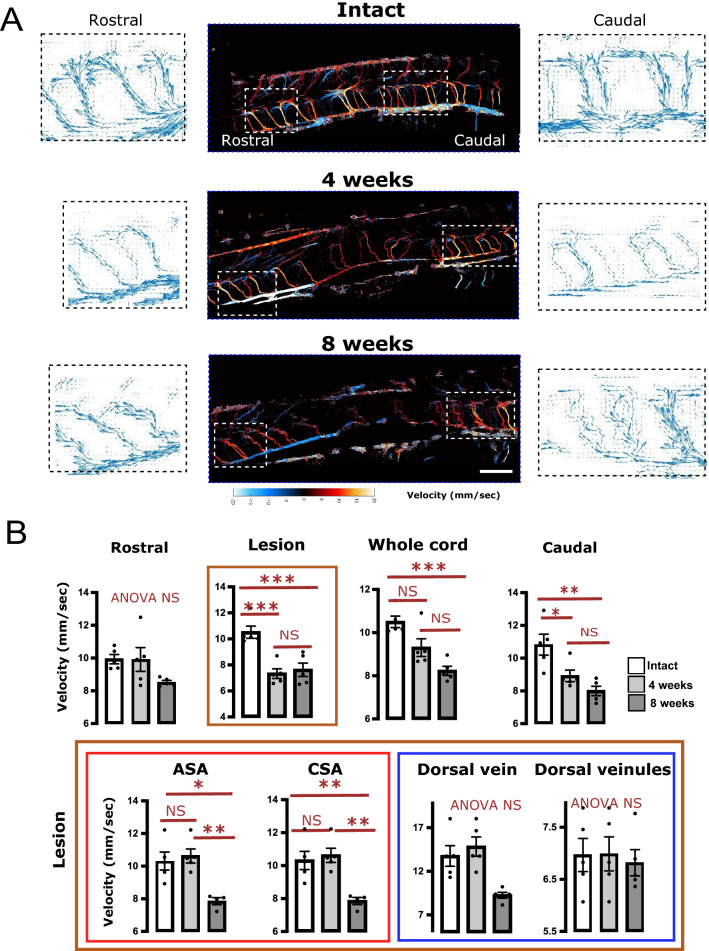
Quantification of bubble velocity using ULM reveals a time-dependent reduction in the blood velocity caudal to, and within the lesion: in ASA and CSA. (**A**) Representative examples of blood velocity (expressed in mm/s, colored central panel) in animals of the different experimental groups: intact, 4 or 8 weeks post-contusion. Examples chosen are the same animals as in Fig. 4. The lateral black and white panels illustrate field vectors of bubble trajectories (i.e. vectors illustrating the local propagation of the bubbles, using both horizontal and vertical velocities) in the rostral or caudal aspect of these examples. The size of the arrows is proportionate to the local speed. These areas are those delineated in white dashed rectangles in the colored panel. These examples illustrate the reduced velocity, associated with increased tortuosity of the vessels (quantified in Fig. 4; here visible at 8 weeks both on the rostral and caudal portions). (**B**) Quantification of the bubble velocity in various vascular compartments. Results are expressed as mean ± SEM and are presented in overlay with individual values. Stats: ANOVA, followed by unpaired t-test as post-hoc test. *p < 0.05, **p < 0.01, ***p < 0.001. NS: Not statistically significant. ANOVA NS: ANOVA *p* > 0.05. The orientations (caudal/rostral) apply to the three examples in (**A**). Bar: 1.3 mm in the central coloured panels and 0.5 mm in the lateral black and white panels.

Second, we quantified changes in blood velocity in the various vascular compartments of intact/lesioned spinal cords by ULM analysis of microbubble speeds (Fig. 5). As shown in examples in Fig. 5A and quantifications in Fig. 5B, the blood velocities are significantly reduced at 4- and 8-weeks post-contusion compared to intact animals, at the lesion site and caudally to it (Fig. 5B, top panels). Here, both the ASA (anterior spinal artery ) and the CSA are affected only at 8 weeks but not at 4 weeks, suggesting a worsening of this alteration at 8 weeks (Fig. 5B, bottom panels). Accordingly, and as highlighted by the examples in Fig. 5A, one striking difference between 4- and 8-weeks post-contusion is the flow reduction within tortuous blood vessels.

### Analysis of vasculature alteration using immunohistochemical staining

**Figure 6:**
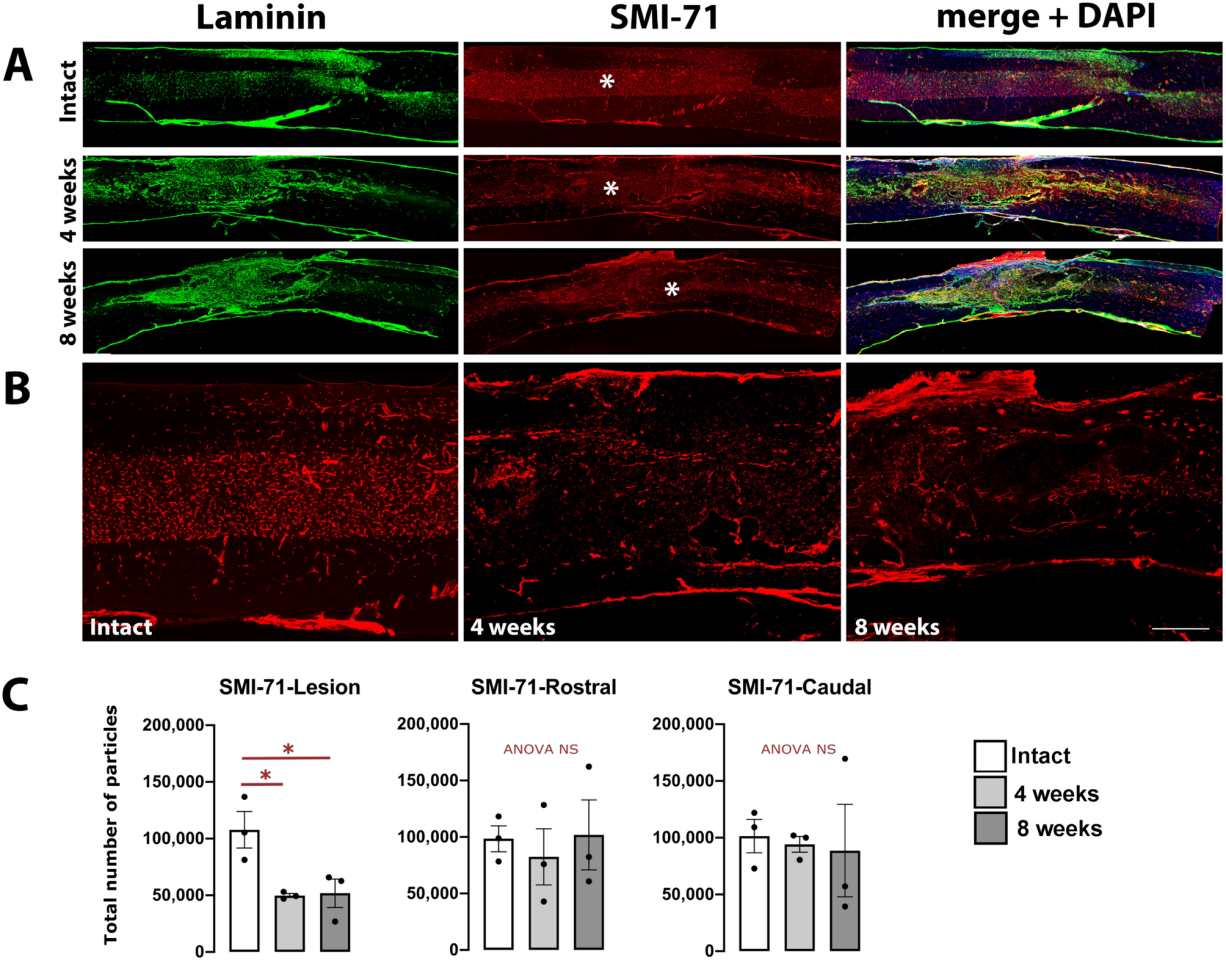
(**A**) Immunofluorescence labeling for laminin (green), SMI-71 (red), and DAPI (blue) on sagittal spinal cord sections from intact (non-injured) rat, and rats at 4- and 8-weeks post-contusion. (**B**) Higher magnification of SMI-71 staining in (**A**) (stars) showing distribution and disruption of blood vessels in parenchymal contused spinal cord. (**C**) Quantification of the number of SMI-71 positive particles in the three experimental groups, in the lesion area and rostral and caudal to it. Quantifications were performed using an identical surface of the cord in all conditions. Bar (**A**): 12 mm, (**B**): 300 µm.

Post-mortem analysis on the same animal was also performed on sagittal spinal cord sections using immunohistochemistry for laminin and for SMI-71, a marker for mature blood vessels^8,11^. While in intact CNS, laminin immuno-labelling reveals basal lamina-associated blood vessels, in traumatic injured SCI, laminin staining was strongly increased at the injury site and in adjacent tissue, revealing the extent of the lesion, as previously described by us and others^8,10,11^ (see Fig. 6A, showing laminin staining from the epicenter towards more distal segments,). The density of mature SMI-71 positive blood vessels was strongly reduced at the injury epicenter (Fig. 6B, center panel). When neo-angiogenesis occurred, from 4- and 8 weeks post-injury (Fig. 6A,B), SMI-71 positive vessels were present within, and also around the primary lesion site, which is often replaced by a cavity. However, the organization of newly formed blood vessel network is disorganized (Fig. 6B) and their density remained significantly lower at the lesion site compared to the intact tissue (Fig. 6C).

### Definition of new biomarkers of vascular dysfunction associated with spinal cord injury severity

**Figure 7:**
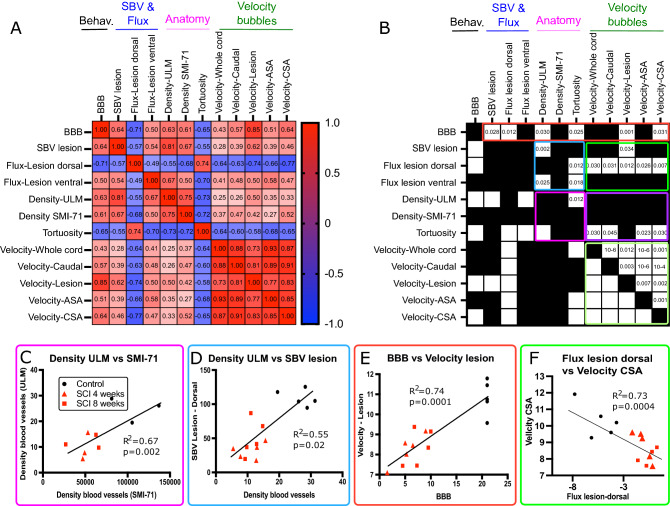
Statistically significant correlations between the locomotor disability of the animals (BBB), changes of SBV within the lesion, main flow, markers of altered vascular anatomy and altered velocity (ULM). Using individual results from all animals included in this study (N = 5 per group), the putative correlation between these different outcome measures were determined using Spearman’s correlation coefficient (**A**), followed by calculation of the corrected *p* value for multiple comparisons (**B**). In (**A**) and (**B**), results are expressed as a double correlation matrix. In A: the color bar indicates the value of positive/negative correlations between these elements. In B: statistically significant cells (corrected *p* value < 0.05) are indicated in white. Black cells are statistically non-significant results. (**A**, **B**) The individual values of either correlation (**A**) or corrected *p* value (**B**) are given in the top half matrix. Six clusters of interest were highlighted in **B**: (1) red: correlation between the BBB and all functional and anatomical measures. (2) Green (bright and pale): correlation between the bubble velocity in various compartments or their correlation with the SBV/ flow. (3) Pink and violet: Correlation between various markers of the vascular integrity (measured both using fUS, ULM and immunohistochemistry) and bubble velocity. (4) Blue: correlations between changes of SBV/flow and the vascular anatomical alterations. Values used for BBB are those of the week of imaging. (**C**–**F**) Four examples of correlations within these measures, showing the individual values in all groups (black circle: control (= non-injured animals); red triangle: SCI 4 weeks and red squares: SCI 8 weeks. Are displayed on each graph the goodness to fit (R^2^) and the *p* value of this single linear regression.

Finally, in order to decipher potential links between these altered biomedical measures that may be relevant for common neuropathological mechanisms associated with SCI and help define biomarkers of vascular dysfunction, we studied the statistical correlations between locomotor behavior, SBV and flow, anatomy of blood vessels, and velocity of bubbles, using individual values from all animals included in this study. Results are presented in a double correlation matrix (Fig. 7), in which in Fig. 7A, the Spearman’s correlation coefficients are displayed. The statistical relevance of these correlations is shown in Fig. 7B (black/white color code; White: statistically significant, *p* < 0.05—Black: non-significant (*p* > 0.05)). The corrected *p* value (correction for multiple comparison using Benjamini–Hochberg test) is indicated in the half top matrix. In Fig. 7B, 6 clusters of statistically significant results were drawn, and Fig. 7C–F present one individual example for each cluster.

These analyses show robustly that: (1) the measure of blood vessel density using ULM correlates very well with the quantification using the ‘gold standard’, i.e. immunohistochemistry of SMI-71 (pink cluster, Fig. 7C), therefore validating the use of ULM for quantification of blood vessel density. (2) Markers of structural vascular alteration also reveal a significant correlation between the reduced velocity of bubbles and the increased tortuosity of the CSA (purple cluster, Fig. 7B). (3) The altered locomotor behavior (BBB) is significantly correlated with the reduction of SBV in the dorsal part of the lesion and with the reduced flow along the cord (red cluster). The BBB was also correlated with the velocity. The lower the BBB (higher impairment), the stronger the reduction in bubble velocity (Fig. 7E). (4) The reduction of SBV within the lesion is associated with the anatomical alterations of the blood vessels (reduced density and increased tortuosity, blue and Fig. 7D). Finally, the reduction of bubble velocity is correlated with the reduced flow in the dorsal horn (bright green cluster and Fig. 7F).

## Discussion

Using multimodal fUS imaging and ULM, this study aimed at investigating in depth the anatomical and structural alterations of the vascular arborization at two time points: 4 weeks post-lesion, a phase coinciding with the restoration of the blood-spinal cord barrier and 8 weeks post-lesion with the establishment of the chronic lesion. Our study provides a quantitative study describing the vascular alterations associated with SCI, with a special interest to both macroscale analysis of the blood flow and its main orientations, but also at microscopic scale, with a quantification of its density, tortuosity and finally speed of blood flow within these blood vessels. The use of these different parameters provides important missing pieces of the SCI puzzle and will help, not only to increase our understanding of the vascular pathophysiological mechanisms underlying SCI, but also to define appropriate biomarkers.

### Alterations in blood volume and blood flow during the establishment of chronic SCI

In contrast with conventional ultrasound imaging, ultrafast ultrasound scanners based on plane wave imaging provide a neuroimaging modality extremely sensitive to displacement of particles, such as red blood cells, but also microbubbles, injected intravenously in ultrasound localization microscopy (ULM). Our first goal was to measure the alterations of SBV and main blood flows in the lesioned cord compared to intact animals.

In agreement with previous angiographic observations (reviewed by^20^) and more recent sensitive ultrasound imaging^12,16,17^, our study confirms a strongly decreased SBV in the lesion site, but also demonstrates a lack of SBV alteration in adjacent segments (both rostral and caudal) that was not reported previously. Interestingly, our approach also reveals that this reduced SBV is highly correlated (*p* = 10^−5^) with the individual locomotor disability of the animals, suggesting a link between motor impairment and the amplitude of hypoperfusion. Indeed, it has been reported that the extent of vascular damage is correlated with the development of secondary lesions after SCI, while neo-angiogenesis plays a key role in the progress of functional recovery after SCI, particularly during the chronic injury phase^7,21^. Accordingly, it has been shown recently that promoting angiogenesis and microvessel density after SCI improves locomotor function recovery^21^.

Furthermore, analysis of the main directional blood flows, quantified here for the first time, brought new, interesting results. Whereas the changes of SBV are restricted to the lesion site, the changes in main directional flow are time-dependent, and widespread along the whole thoracic cord. Interestingly, unlike changes of SBV, the reduction of top-down flow along the dorsal thoracic cord, that is worsening at 8 weeks, is linked to the altered vascular morphology in the ventral horn (tortuosity) and the reduced velocity of microbubbles, and these parameters are linked statistically. These results suggest that to assess functional integrity of the spinal blood flow, the measure of the flow directionality is more sensitive than the local measure of SBV. These subtle alterations may be due to the observed anatomical alterations in the arteries (ASA, CSA), subsequently leading to a reduced blood flow in the arteries vascularizing the dorsal horn.

Previous studies measuring spinal blood volume alterations following SCI were mainly performed at very early time points (i.e. within hours/days post injury), where the decreased blood volume is due to the initial hemorrhage, followed by spinal ischemia. The grey matter naturally receives the largest blood supply compared to white matter due to its dense network of capillaries. As previously discussed^22^, ischemia in the grey matter therefore leads to a quick and widespread cell death, necrosis, debris formation, rapidly followed by neuroinflammation and cavitation. After the largely documented early decrease in the density of blood vessels^7,11,23–27^, an adaptive vascular response takes place with angiogenesis and re-opening of the microcirculation^7,23,24,28^. The time points chosen in our study (4 and 8 weeks post-injury) up to the establishment of the chronic phase, encompass the formation of new blood vessels, but also necrotic cavities. Interestingly, several of our measurements of the spinal structural and functional vasculature integrity (reduced arterial velocity, inverted flow in the ventral horn) showed a worsening between 4 and 8 weeks post-contusion, probably due to the highest progression of secondary lesions leading to cavitation. Indeed, from 4 weeks post-injury on, the immune response becomes a rather persistent inflammatory state. Such environment affects the autonomous tissue repair, including axonal plasticity initiated in the sub-acute phase, but largely aborted in the course of tissue inflammation and necrosis^29,30,31^.

### Invaluable contribution of ULM for the estimation of blood vessel density, speed of micro-bubbles and blood vessel density

in the field of pre-clinical neuroimaging of the lesioned spinal cord, microbubbles were used in the past simply as contrast agents^16,17^. Here, these microbubbles were used differently. We previously demonstrated that in the brain, by imaging at a fast framerate, it is possible to detect individual microbubbles. Thus, microbubbles allowed us to visualize in live animals the fine structure of blood vessels at the microscopic (10 μm) scale, an approach termed ‘ultrasound localization microscopy’ (ULM)^32^. Tracking of these microbubbles, on the other hand, enables us to measure particle speed, equivalent to local blood velocity, at the same microscopic scale. More recently, we demonstrated that ULM is applicable to the lumbar spinal cord^15^ in intact animals. In the present study, we went one step further, using the invaluable spatial resolution and sensitivity of this technique on lesioned spinal cord to quantify structural damage to the vasculature and changes in blood velocity.

Furthermore, our approach allowed for detailed quantitative measurements of the blood velocity in sub-parts of the damaged vascularization. The speed of blood flow observed in the lesioned spinal cord is consistent with a previous report by Khaing et al. on early stages post-injury using Contrast Enhanced Ultrasound^16^. We convincingly show a massive reduction in blood velocity within and caudal to the lesion at both 4 and 8 weeks post-contusion. Because these changes were also observed in the local arteries (ASA and CSA) that provide 2/3 of the vascularization in the ventral horn^33^, we suggest that the observed effect in the lesion site is due to a reduction in the blood flow in these arteries. As previously quantified using micro-computed tomography^26,27^, and confirmed here, the shape of the CSA is altered, giving rise to a non-orthogonal ascending flow. The number of branches of the CSA and its diameter are also reduced^26,34^. Our statistical analysis proves that these alterations are correlated with increased tortuosity. It is indeed likely that these structural alterations are the cause of the decreased blood velocity.

Finally, these changes come along with a dramatic reduction of local blood vessel density within and caudal to the lesion at both 4- and 8 weeks post-contusion, as demonstrated both by ULM measurement of the density of blood vessels, and by immunohistochemical quantification of blood vessels in fixed spinal cord of the same animals. Both approaches provided similar, statistically equivalent results, validating the use of ULM for the quantification of structural vascular alterations. Moreover, the observed reduced blood vessel density is consistent with previous reports on hemorrhage and vascular plasticity^26^.

### Rostro-caudal asymmetry of the vascular alterations

So far, only few studies investigated the anatomical and functional damage following SCI by comparing the alterations occurring rostrally versus caudally from the initial lesion site. Strotton et al.^22^, in a thorough spatio-temporal 3D contrast micro-computed tomography (CT) study, elegantly showed the structural alterations in spinal grey and white matters and dorsal columns. They reported that although rostral and caudal adjacent segments undergo similar alterations, their magnitude is significantly higher caudally than in rostral segments. This is particularly true for the vasculature damage^35^, as also demonstrated by our present study. Thus, we found significantly reduced blood velocities in caudal segments compared to intact animals, as well as a tendency for SBV reduction. The pronounced vasculature damage in caudal segments appeared to be related to the unexpected chronic hypoxia in the cord far caudal of the injury epicenter that has recently been described^35^. This study also provided a mechanism that underlies such rostro-caudal asymmetry of vasculature alteration: even months after SCI, the spinal cord below the site of injury remains in a chronic state of hypoxia owing to paradoxical excessive activity of monoamine receptors (5-HT1) on pericytes, despite the absence of monoamines. This monoamine receptor activity causes pericytes to locally constrict capillaries, which reduces blood flow to ischemic levels. Inhibition of monoamine receptors, or increase in inhaled oxygen, produces substantial relief from hypoxia and improves locomotor function recovery. Here, using ULM, our study confirms the strong asymmetry in blood speed between rostral and caudal segments, suggesting that the underlying mechanisms, previously described for 6 months post-lesion^35^, are active much earlier, from the establishment of the chronic lesion (here shown at 4 and 8 weeks post-injury).

### Towards patient’s stratification using UDI and ULM

Results brought forward in this study were obtained in one of the most clinically-relevant animal models of SCI, that exhibits several neuropathological outcomes seen in patients with SCI^36^. The most important of which are systemic and local vascular insults, electrolyte shifts, oedema and excitotoxicity. These secondary processes contribute to the evolution of the pathological changes which when severe, progress from central hemorrhagic necrosis involving mainly the grey matter, but also white matter at the injury site. As in human SCI, this results in cavity formation at the injury site routinely seen in human SCI and also in rat (but not in all mouse strains) experimental models that increases in size rostrally and caudaly with lesion chronicity. Importantly, the common feature in all experimental models and in human cord injury is the early hemorrhage in the central region of the injured cord, especially in the grey matter. It is thus, very likely that vasculature changes and remodeling described in our present study also occurs in human after SCI. Therefore, for SCI pathophysiology, reliable prognosis instruments are critically needed, be it for the individualized neurological treatment of patients, or the selection of patients for clinical trials. Based on age and clinical neurological parameters (with or without imaging, depending on the studies), several teams provided prognostic models of the patient’s independent walking^37,38^, or urinary continence, 1 year after SCI^39^.

In order to go further, the identification and validation of early biomarkers of the degree of neural and vascular damage, predictive of the neurological outcome, is under active investigation. Current biomarkers include imaging readouts of neural alterations, and titrations of particular biomolecules in the cerebrospinal fluid or in the serum of patients (see for review^3,40^). The early extent of the hemorrhage and the degree of vascular alteration play a determinant role in the patients’ functional recovery. Inclusion of the measurements at a very early stage, i.e. during decompression surgery (when the spinal cord is directly accessible) and possibly later, transcutaneous^17^ if the materials inserted allows ultrasound imaging, would provide accurate information on vascular alterations, including reduced flows in the different spinal vascular compartments.

We previously showed that UDI and ULM are applicable to human brain, both non-invasively in neonates^41^ and adults^42^, and also during perioperative interventions in adult patients^43^. The precise analysis of vasculature state, along with other biomarkers previously described (blood serum cytokines, MRI, DTI^44–47^) would provide a more complete picture of the pathophysiological changes in patients with various degrees of injury severity, and allow for a refined/more accurate prognosis in view of the long-term follow up of these patients.

## Materials and methods

All experiments performed in this study were in accordance to the French and European Community Council Directive of September 22 (2010/63/UE). They were also approved by the local Institutional Animal Care and Ethics Committees (#59, ‘Paris Centre et Sud’ project #2018-05 and Sorbonne University project #1514.01). Accordingly, the number of animals in our study was kept to the minimum necessary. We established in a preliminary experiment that N = 5 was the smallest number of animals required per group to detect statistical difference in our imaging experiments. Finally, all methods are in accordance with ARRIVE guidelines.

### Surgical preparation and SCI model

Animals arrived in the animal facilities at the IBPS institute 2 weeks before the beginning of experiments. Fifty adult female Wistar rats (225–250 g) were obtained from Janvier labs (France) and housed under controlled temperature (22 ± 1 °C), relative humidity (55 ± 10%) and 12 h light–dark cycle. Before and after surgical interventions food and water were available ad libitum.

Surgical procedures were performed under reversible, continuous Isoflurane anesthesia (2–2.5%, Isofluran^®^) and sterile precautions were used throughout. In order to perform the spinal cord contusion, skin and musculature was cut from T7–T10 and the dorsal surface of T8–T9 exposed by laminectomy, with the dura remaining intact. The vertebral column was stabilized by fixing vertebral bodies rostrally and caudally to the impact area with clamps attached to the base of the Benchmark^™^ impactor device. A 2.5 mm diameter tip was delivered from 7 mm height with a speed of 1.96 m/s over the spinal cord dorsal surface, and contact time after the impact was 1 ms. These parameters lead to a severe lesion with pronounced locomotor deficits evaluated using the Basso, Beattie and Bresnahan locomotor rating scale (initial BBB score 1 day after the trauma between 0–0.5).

To prevent urinary infections, rats received subcutaneous injections of eurofloxacin (Baytril^®^ 10%) once a day during the first week post-lesion. Until restoration of normal micturition, bladders were manually emptied twice a day, and the health state of operated rats monitored by regular weight and visual inspection of the surgical wound.

The rats were randomly assigned into three groups, five animals per group: intact (received a sham lesion, where the muscles were opened but the spinal cord left intact), 4- and 8-weeks post-injury. BBB locomotor scores were taken at days 1, 7, and then weekly for a total of 7 weeks. Since the animals were transported to another institute for spinal cord imaging, recording of BBB scores was stopped 1 week before the experiment. At the end, the rats were sacrificed for histological investigation.

### Surgical procedures and preparation for imaging

Under deep anesthesia (IP) bolus of Medetomidine (Domitor, 0.4 mg kg^−1^) and ketamine (Imalgène, 40 mg kg^−1^), a laminectomy (centered on the spinal cord lesion) was performed between thoracic T6 and lumbar L2 vertebrae (two vertebrae above and below the initial laminectomy), thus opening a window allowing positioning of the entire ultrasonic probe (14 mm) in a sagittal plane.

Thereafter, the animal was placed on a spinal cord stereotaxic frame. Anesthesia was maintained but reduced, using subcutaneous perfusion of Medetomidine (0.2 mg/kg/h) and ketamine (25 mg/kg/h) using a syringe pump. During the surgical procedure and the imaging session, the animal’s body temperature was kept at 37 °C using a heating blanket and an intrarectal probe (Physitemp, USA), and heart and respiratory frequencies were monitored (MouseOxPlus, Ugo Basile, Italy). Each imaging session lasted 2–3 h.

### Ultrafast Doppler imaging and signal analysis

Two milliliters of saline were gently dropped on the spinal cord (the dura mater was kept intact), and the window created by laminectomy was then filled with echographic gel. The ultrasonic probe (*f* = 15 MHz, 100 µm spatial pitch, 128 elements, Vermon, France) was positioned just above the window using a 3-axis motor. The probe connected to an ultrasonic ultrafast imager (Iconeus, 128 channels, 62.5 MHz sampling rate) was driven with a prototype software (Iconeus, Paris, France, and Inserm Accelerator of Technological Research in Biomedical Ultrasound, Paris, France). The imaging session started by a 3D scan of the spinal cord that allowed positioning of the probe. By symmetry of the vascular structure, the median plane was deduced and the alignment of the probe with the spinal cord adjusted by rotating it until the anterior spinal artery (ASA) was entirely seen on the images. Ultrafast Doppler at 5500 Hz PRF for relative CBV, Ultrafast Doppler at 20,000 Hz PRF for blood flow direction and at 5000 Hz PRF for ultrasound localization microscopy acquisitions were performed successively.

#### Measure of SBV using Power Doppler imaging

SBV values were obtained with the ultrafast Doppler imaging method, which consists of compounded plane-wave ultrasound transmissions^48^. Thus, each frame was a compound plane wave frame resulting from the coherent summation of 11 compounded tilted plane waves with angles separated by 2° and ranging from − 10° to 10°. The framerate used was 500 Hz, resulting in a 5500 Hz pulse repetition frequency (PRF). The Singular Value Decomposition (SVD) clutter filtering (Demene et al. 2015) typically used for brain tissue had to be modified for our use on spinal cord tissue. The SVD thresholds were automatically determined thanks to adaptive spatiotemporal SVD using the similarity of spatial singular vectors^49^. Over 400 compounded frames, the first 30 singular values were excluded to remove the tissue and the last 230 were excluded to remove the noise. The resulting 400 successive images were averaged to obtain a single SBV image that was analyzed by spatial averaging of the different ROI drawn using MATLAB 2020a.

#### Choice and size of areas taken for the measurements of SBV, directions of flow, density of blood vessels and bubble velocity

For the quantification (of the SBV, density of blood vessels and bubble velocity) in the lesion, rostral and caudal to the lesion: three subparts of equivalent area (30 mm along the antero-posterior axis X 18–20 mm dorsal to caudal, i.e. 530 mm^2^) of the thoracic spinal cord were drawn: at the lesion site (around the lesion epicenter), rostral or caudal to it. In intact animals, the same areas were taken, the area at the center was taken for the counter part of the lesion side, and, as in lesioned animals, the same 530 mm^2^ rostral and caudal to the center were taken for the rostral and caudal values. The respective values were computed on MATLAB.

Quantifications of bubble velocity in the ‘whole cord’ (Fig. 5), an equivalent rectangle surrounding the entire thoracic cord imaged (or most of it) of the following dimensions 90 mm (Rostro-caudal) X 18–20 mm (dorso-ventral axis) was computed on MATLAB in all animals included in our study.

The same principle was used for all the other measures: an equivalent area was defined in all groups, surrounding either the dorsal/ventral thoracic cord (Fig. 3), CSA, ASA, dorsal vein (Figs. 4, 5).

#### Direction of flow

A different acquisition sequence was used to analyze the direction of flow. Each frame was a compound image of 5 angles (− 4°, − 2°, 0°, 2° and 4°), which together with a sampling frequency increased to 4000 Hz resulted in a 20,000 Hz PRF. The total acquisition contained 2000 frames and lasted 0.5 s. The same SVD filter as for the Power Doppler acquisition was used to separate the tissue signal from the blood signal. Using the same spectral analysis as in the color Doppler images^50^, the SBV signal was separated into two series of images representing the SBV going toward and away from the probe. After averaging the 2000 frames of the two directional SBV, the general directional SBV was obtained by subtracting the SBV going upward from the SBV going downward, creating a directional power image. The images were then spatially averaged in the same ROIs drawn for the analysis of the SBV previously described.

#### Ultrasound localization microscopy (ULM)

A catheter filled with saline was inserted in the rat jugular vein before positioning the animal on the stereotaxic frame. After all previously mentioned measures, ULM was performed similarly to the methods described in^42^ using the same ultrasound imager as above. Compounded frames were acquired at a 1000 Hz framerate (with angles at − 4°, − 2°, 0°, + 2°, + 4°, PRF = 5000 Hz) using the same system (Iconeus One, Iconeus, Paris, France) as above for a total time of 150 s. Beamformed data were filtered using the SVD spatio-temporal filter described in^51^, and the 7 first singular values were removed to extract microbubble signals from the surrounding tissues. Microbubbles were detected as the brightest local maxima in the images. Tracking of the maxima positions was performed using the Iconeus software (Iconeus, Paris, France, and Inserm Accelerator of Technological Research in Biomedical Ultrasound, Paris, France) and gives all positions in space (x-axis and z-axis positions) and time (frame number) for each detected individual bubble.

The successive positions gathered in one track were used to compute the interframe bubble velocity vector components (along probe x-axis and depth z-axis), and absolute velocity magnitude.

Density maps were computed by counting all the microbubbles that passed through each pixel during acquisitions. Since the total number of bubbles injected is not exactly the same from one injection to another, comparison of raw density between animals would reflect this random effect instead of intrinsic differences between groups. To avoid this variability, the value of density was normalized by the total number of bubbles detected in the ROI contouring the whole spinal cord. To compare these maps between animals and consider the variability coming from the random number of bubbles injected into the blood stream, the value of density was normalized by the total number of bubbles detected in the spinal cord imaged.

Analysis of the density of blood vessels using the ULM analysis was performed using Image J. The images obtained at the previous steps were first transformed as black and white pictures. Using a common threshold (131) for all animals, the percentage of staining occupied by the blood vessels was measured in each animal in the six following compartments: Lesion site, rostral or caudal to the lesion site, the ASA or the CSA at the level of the lesion site. For each compartment, the area measured for all animals was similar. Results are expressed as percentage of area occupied by the staining.

The vessel tortuosity index was computed thanks to the MATLAB function “vessel_tortuosity_index” written by Maz M. Khansari^52^. For each animal, three vessels located rostral, caudal and the last one close to the lesion, were chosen on the density map given by the ULM algorithm. A line was drawn over each one to use it as an input for the function. These values were averaged to obtain the tortuosity index for each animal.

Each pixel of the velocity maps was computed as the mean velocity of every micro-bubble detected in this pixel during the whole acquisition. These two maps were then spatially averaged in ROIs drawn the same way as for the SBV analysis.

#### Statistical analysis

For each type of statistical comparison, the same protocol of hypothesis verification was applied to choose the appropriate statistical test. First, the two assumptions needed for parametric tests were tested. The normality of the data distribution of each group was tested with a Lilliefors test and the equality of the variances was tested with a Bartlett’s test.

Then, an ANOVA was performed to determine if a difference between two groups could be detected with a statistical test. If one of the two conditions for the parametric tests was not met, the Kruskal–Wallis test was performed for the same purpose. If the ANOVA detected a statistically significant difference, pair wise tests were used to determined which groups differ. For data suited for parametric test, a student test was used. A Wilcoxon rank sum test was used otherwise.

In Fig. 7, for the double correlation matrix: Considering that some measures did not have a gaussian distribution (and were therefore not compatible with a parametric test), all correlations were computed using Spearman coefficients, (which is the non-parametric counterpart of the Pearson’s Linear Correlation Coefficient). The *p* values corresponds to the probability of rejection of the null hypothesis that the correlation is equal to zero. All panels of Fig. 7 were plotted using GraphPad.

Statistical analysis was performed using Matlab. Graphs were made using GraphPad.

### Perfusion and immunohistochemistry

At the end of the experiment, rats were administered a lethal dose of pentobarbital (Euthasol^®^ 400 mg/mL; 150 mg/Kg), and were transcardially perfused with saline 9‰ at 37 °C, then with 4% paraformaldehyde (PFA) in 0.1 M phosphate buffer. PFA-fixed tissues were incubated in 15% and 30% sucrose for 24 h, and 48 h at 4 °C, respectively, then embedded in optimal cutting temperature compound (OCT; Tissue-Tek), quickly frozen in a dry ice/isopentane bath and stored at − 80 °C. 40 μm sagittal sections were cut on a cryostat (Leica CM3050 S) and mounted on glass slides (Superfrost^®^ Plus).

For immunolabeling, sections were permeabilized with 0.3% Triton X_-100 in PBS during 5 min at room temperature, and incubated for 1 h in 10% bovine serum albumin (BSA)/PBS. Blocking was followed by incubation overnight at room temperature with the primary antibodies diluted in 5% BSA/PBS. The following primary antibodies were used: rabbit glial fibrillary acidic protein (GFAP; Dako, 1:2000), mouse-SMI 71 (Covance, 1:100), rabbit-a-laminin (Sigma, 1:100). Finally, appropriate secondary antibodies (Alexa Fluor 488 and 555 conjugated, 1:1000; Life technologies) diluted in BSA 5% were used.

Figures showing large longitudinal spinal cord sections were produced using a Zeiss Axio Zoom V16 microscope with image stitching and were processed using ImageJ (NIH, USA).

#### Quantification of SMI-71 positive cells

Pictures of 4 sections/rat (160 µm between each section) stained with anti-SMI 71 antibody were taken for quantification. With ImageJ, a rectangle of 1.6 mm^2^ was drawn at the level of lesion, as well as at the level of the caudal and rostral part of each section. After fixing the same threshold for each section, particle analysis was performed and the mean value per rat was calculated and compared as total area between each group (intact-uninjured, 4 and 8 weeks post-injury; 3 rats per group). One-way Anova was done to compare values for each region between groups.
